# The *klotho* F/C AD risk haplotype drives distinct phenotypes in a novel mouse model

**DOI:** 10.1002/alz.090967

**Published:** 2025-01-03

**Authors:** Michael Sasner, Ravi S Pandey, Kevin P Kotredes, Dylan Garceau, Channing Weber, Rafiou Agoro, Christoph Preuss, Gregory W Carter

**Affiliations:** ^1^ The Jackson Laboratory, Bar Harbor, ME USA; ^2^ The Jackson Laboratory for Genomic Medicine, Farmington, CT USA

## Abstract

**Background:**

Apolipoprotein E4 (APOE4), a common variant of *APOE*, is a major genetic risk factor for Alzheimer’s disease (AD), but APOE4 carriers do not always develop AD. Several large‐scale genetic studies have identified a common haplotype of the aging factor *klotho* that modify disease risk in APOE4 carriers. In humans, *klotho*harbors two common missense variants (rs9536314, p.F352V; rs9527025, p.C370S) that define the klotho V/S (KL‐V/S) haplotype, which is protective against AD in APOE4 carriers, and the klotho F/C (KL‐F/C) haplotype, which is not. We sought to understand this genetic interaction using novel mouse models.

**Method:**

Mouse *klotho* differs at the key amino acid position p.C370S rendering the native mouse haplotype as “KL‐F/S”, matching neither of the observed human haplotypes. We have replaced this with either the homozygous protective KL‐V/S or common KL‐F/C haplotypes using CRISPR/Cas9. We have tested the effects of these *klotho* haplotypes on klotho expression in serum, brain, and kidney relative to B6 controls and to the amyloidogenic App^SAA^ AD model, which exhibit significant amyloid neuropathology. We have also run RNA‐seq on whole brain homogenates at 12 months of age.

**Result:**

Secreted p‐KL was decreased in KL‐F/C mice harboring the novel mouse risk haplotype relative to age matched B6 controls and to KL‐V/S mice. In contrast, KL‐V/S mice showed no significant differences in soluble p‐KL serum levels when compared to B6 controls. Secreted p‐KL levels in KL‐V/S mice were significantly elevated when compared to the APP^SAA^ mice, whereas young KL‐F/C mice showed similar levels to the aged APP^SAA^ model. Transcriptional analysis indicated that the KL‐V/S haplotype was anti‐correlated with AMP‐AD human modules, while the KL‐F/C risk haplotype was significantly correlated to AD signatures in modules enriched for neuronal system, cell cycle, myelination, and cellular stress response pathways.

**Conclusion:**

These data suggest the native mouse *klotho* allele may act similarly to the protective KL‐V/S haplotype, while the KL‐F/C haplotype leads to lower secreted klotho and potentially greater disease risk. These models are therefore suitable for experiments to elucidate how *klotho* variants protect from AD pathologies, and will enable detailed studies of how this protection is specific to the APOE4 genotype.

